# The Temporal Relationship between Selected Mental Disorders and Substance-Related Disorders: A Nationwide Population-Based Cohort Study

**DOI:** 10.1155/2018/5697103

**Published:** 2018-10-04

**Authors:** Mu-Lin Chiu, Chi-Fung Cheng, Wen-Miin Liang, Pen-Tang Lin, Trong-Neng Wu, Chiu-Ying Chen

**Affiliations:** ^1^National Institute of Infectious Diseases and Vaccinology, National Health Research Institutes, Zhunan, Taiwan; ^2^Department of Public Health, China Medical University, Taichung, Taiwan; ^3^Graduate Institute of Biostatistics, China Medical University, Taichung, Taiwan; ^4^Psychiatric Department, Taichung Veterans General Hospital, Taichung, Taiwan; ^5^Department of Healthcare Administration, Asia University, Taichung, Taiwan; ^6^Graduate Institute of Clinical Medical Science, College of Medicine, China Medical University, Taichung, Taiwan

## Abstract

*Introduction. *Previous studies have examined the association between specific mental disorders, particularly mood and anxiety disorders, and substance-related disorders; but the temporal link between them remains unclear. This study aimed to examine whether individuals with specific mental disorders, including affective psychoses, neurotic disorders, schizophrenia, personality disorders, and adjustment reaction, have higher risks for subsequently developing substance-related disorders compared to those without.* Methods. *A large-scale study with longitudinal data was conducted using the Taiwan National Health Insurance Research Database (NHIRD) consisting of 2,000,118 patients' medical records from 2000 to 2009. A total of 124,423 people diagnosed with selected mental disorders and the same number of people without the diagnoses of the selected disorders were identified between January 1, 2001, and December 31, 2006, and followed up for the diagnoses of substance-related disorders till the end of 2009. We estimated the risk for subsequently developing substance-related disorders among patients with the selected mental disorders compared to those without by using Cox proportional hazard models. The cumulative incidence of substance-related disorders was calculated using the Kaplan-Meier method.* Results. *The risk for developing substance-related disorders in patients with selected mental disorders is about 5 times (HR=5.09, 95% CI: 4.74-5.48) higher than those without after adjusting for potential confounding variables. From the multivariate analyses of subsamples stratified by age, sex, and urban and income levels, we found all adjusted hazard ratios were significantly higher than 1.0, ranging from 2.12 (95% CI: 1.72-2.62) to 14.55 (95% CI: 7.89-26.83). For children and adolescents aged 10-19 years, those with specific mental disorders had 14.55-fold higher risk for developing substance-related disorders in later life compared to their counterparts. Furthermore, patients with personality disorders had the highest risk (HR=25.05).* Conclusions. *The earlier onset of the selected mental disorders is a potential risk for developing substance-related disorders in later life, particularly for personality disorders. Health professionals should pay more attention to this at-risk population, especially to adolescents with mental disorders.

## 1. Introduction

Substance-related disorders (SRDs), comprised of substance use disorders (SUDs) and substance-induced disorders (SIDs), are disorders of intoxication, dependence, abuse, and substance withdrawal caused by various substances, both legal and illegal. According to the 2012 World Drug Report made by the United Nations Office on Drugs and Crime (UNODC), every year 2.3 million, 5.1 million, and 250,000 people die from alcohol, tobacco, and drug-related illnesses, respectively. Among the global population aged 15 years and older, the annual prevalence rate for alcohol use is 42%, for tobacco use is 25%, and for illicit drug use is 5% [1]. The subsequent negative effects of these substance-related problems on population health and increasing economic and social burdens have become an important issue in global health [2, 3].

The coexistence of mental disorders with substance use or substance-related disorders has been well documented [4–7]. For example, the estimated U.S. population lifetime prevalence of comorbid alcohol and drug disorder for adults with a mental disorder was 29%. Notably, the prevalence rates of comorbidity of any of mental disorders for adults with any addictive disorders (36.6% for alcohol; 53% for drug disorders) were all higher than the aforementioned comorbidity rate [4].

Existing studies have further examined the temporal relationships between psychiatric disorders and substance disorders or substance use in understanding the etiology among the disorders, but the link was inconclusive. Some indicated that earlier substance use or SUDs could increase the risks for the subsequent onset of depressive, bipolar, and anxiety disorders [8–14], while others indicated contrasting results [5, 15–21]. These contrasts may be due to the variations in the methodology such as study design, sample selection, and onset measurement and due to the limitations from small sample sizes or short follow-up periods across the studies. Most longitudinal studies with retrospective data encounter recall bias from self-report information [5, 9–16]. Several studies lack information on childhood and early adolescence [9–13]. Some studies are limited to small sample sizes [15, 22], while others with large sample sizes are not based on community populations or not nationally representative [14, 16, 22]. However, recent studies have suggested that an underlying susceptibility to mental disorders causes people to use substances to self-medicate the subthreshold symptoms of their incipient mental disorders, resulting in the full diagnosis of SUDs [21–23]. Hence, the temporal link between mental disorders and substance-related disorders requires clarification.

A body of literature indicated that mental problems occurring in childhood and adolescence could increase the risk for substance use and/or abuse in adulthood [24–27]. In addition, among the empirical studies we found more research considering mood disorders, anxiety disorders, personality disorders, and conduct disorders as risk factors for SRDs than schizophrenia or other psychiatric diseases [5, 7, 15, 22, 28]. Therefore, the primary interest of our study is to examine the temporal relationship of the specific mental disorders including affective psychoses, neurotic disorders, personality disorders, and adjustment reaction as well as schizophrenia with the later onset of substance-related disorders by using data with follow-up medical records from a large population-based sample of more than two million individuals, which allowed us to ascertain the diseases' diagnoses and their temporal links. Previous studies have indicated that characteristics such as age [29–31], sex [32–35], education level [36], income and employment [37, 38], and socioeconomic status [39–44], as well as urban and rural status [45–48], all have effects on the rate of substance use or abuse. Therefore, we adjusted for these potential confounding variables to investigate the temporal pattern of the onset of the above-mentioned mental disorders prior to SRDs by adopting a random sampling strategy with matching the characteristics for those with the selected mental disorders in comparison to those without.

## 2. Methods

### 2.1. Data Source

We utilized a database of 2,000,118 beneficiaries, randomly sampled from the registration data of all beneficiaries enrolled in the Taiwan National Health Insurance Program (TNHIP) in the year 2000. Individual medical records were followed up till the end of the year 2009, and available records could be traced back to the beginning of year 1995 of TNHIP, namely, the National Health Insurance Research Database (NHIRD). This is a nationally representative sample managed and provided by the Collaboration Center of Health Information Application (CCHIA) at the Ministry of Health and Welfare of Taiwan. The enrolled beneficiaries cover 99.9% of over 23 million people of Taiwan [49]. NHIRD is widely used because of its large-scale and longitudinal advantages. As the database consists of de-identified secondary data for research purposes, this study was exempt from a full ethics review and instead only required a basic one approved by the Institutional Review Board at China Medical University Hospital.

### 2.2. Study Design

We conducted a retrospective cohort study and observed two cohorts, one with a selected mental disorder and one without, and traced their medical outcome of having been diagnosed with a substance-related disorder. The cohort of beneficiaries with selected mental disorders consisted of people diagnosed with schizophrenia, affective psychoses, neurotic disorders, personality disorders, or adjustment reaction in accordance with the International Classification of Diseases, Ninth Revision, Clinical Modification (ICD-9-CM) codes of 295.xx, 296.xx, 300.xx, 301.xx, or 309.xx, respectively, from January 1, 2001, to December 31, 2006. To increase the validity of clinical diagnoses, mental disorder classification was defined as having a history of at least three outpatient visits within one year or a one-time hospitalization. The first day a person is diagnosed with any one of the selected mental disorders identified from at most three ICD-9-CM codes (in addition to the principal diagnostic code, the secondary and tertiary diagnostic codes were considered if they existed) was defined as the index date. The cohort of the control group was randomly selected from those beneficiaries without selected mental disorders with matching by age (±3 years), sex, urban level, income level, and index date at a ratio of 1:1 (case:control) within the same period. The levels of urbanization were categorized according to the stratification of Taiwan Townships developed by Liu et al. [50]. Six income levels were categorized according to the insurance amount classified by the National Health Insurance Administration, Taiwan. Subjects who were diagnosed with substance-related disorders before the index date, on the index date, and one year after the index date were all excluded from our study.

The substance-related disorders (SRDs), including alcohol-induced mental disorders, drug-induced mental disorders, alcohol dependence syndrome, drug dependence, or nondependent abuse of drugs, were identified in accordance with the ICD-9-CM codes of 291.xx, 292.xx, 303.xx, 304.xx, or 305.xx, respectively. And we also defined substance-related disorders as having the diagnostic codes in at least three outpatient visits within one year or a one-time hospitalization from January 1, 2001, to the end of December 31, 2009. Finally, we obtained a total of 124,423 subjects with selected mental disorders and the same size of control subjects. Among the two cohorts, for each matched pair, the period of time after the index date was set as the entire observational period, and we followed up until the occurrence of substance-related disorders, death, drop out from the insurance system, or the end of 2009. Therefore, each subject was followed for at least 3 years.

### 2.3. Statistical Analysis

To determine whether specific mental disorders are the antecedents to substance-related disorders, the study estimated their hazard ratios (HRs) adjusted for age, sex, and urban and income levels using the Cox proportional hazard models. Furthermore, the model was also used to analyze subsamples stratified by age, sex, and urban and income levels to examine relationships across subpopulations. The Kaplan-Meier method was used to calculate the cumulative incidence of substance-related disorders in subjects with selected mental disorders and those without. The model was also used for obtaining the estimated risks of each subtype of SRDs for the selected mental disorders as compared to their counterparts by adjusting for age, sex, and urban and income levels. Before conducting the above analyses, the comparison of the baseline characteristics between two cohort subjects was made using a *χ*^2^ test for categorical variables. All data management and calculation of HR and 95% Confidence Interval (CI) were performed using the SAS System (version 9.3; SAS Institute, Cary, NC). Results with a p value less than .05 were considered statistically significant.

## 3. Results

**Figure 1:**
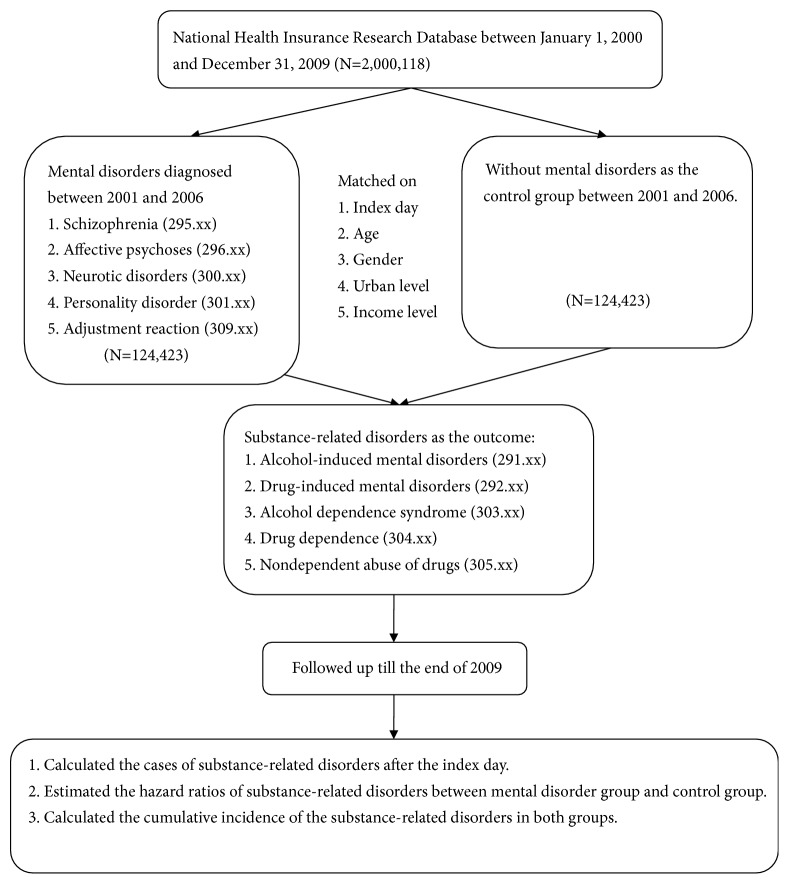
Study flow.

From the longitudinal health insurance database, this study identified 124,423 patients clinically diagnosed with selected mental disorders between 2001 and 2006. Simultaneously, this study randomly selected 124,423 people without selected mental disorders as the control cohort matched to each case's index date, age, sex, and urban and income levels after excluding subjects who had SRDs before, on, and one year after the index date. The mean follow-up duration was 6.19 (±1.75) years.  Figure 1 shows the details of the process of selecting study subjects. Due to our matching process, the distributions of age, sex, urban level, and income level were similar at the baseline between the cohort subjects with selected mental disorders and those without. Among the subjects with the selected mental disorders, the majority of them were diagnosed with neurotic disorders (91.71%), followed by affective psychoses (17.48%), adjustment reaction (4.93%), schizophrenia (2.65%), and personality disorders (1.37%) in descending order (Table 1), suggesting that neurotic disorders were the most prevalent comorbid diseases, followed by comorbid affective psychoses.

**Figure 2:**
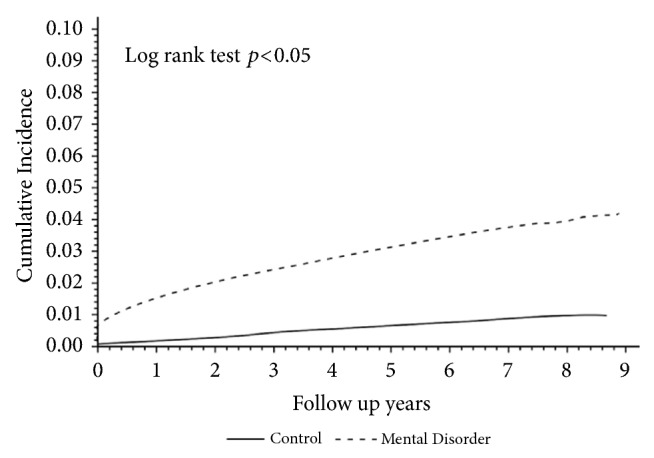
Cumulative incidence of substance-related disorders in patients with mental disorders compared to those without.

Table 2 shows the follow-up profiles for the two groups. The median duration from the index date of the diagnosis to the subsequent onset of SRDs in the mental disorder group was shorter than that in the control (1.54 < 3.22 years), and the difference in the mean duration was significant (2.12 < 3.28 years, *p* < 0.001). The proportions of the subsequent SRD cases and death during the follow-up period in the mental disorder group were more than that in the control (3.41% > 0.69% and 8.34% > 6.9%, respectively). The proportion of the loss of follow-up in both groups was similar and few (0.17% and 0.16%). It is interesting to note that the major subtype of SRDs was the nondependent abuse of drugs in both the mental disorder group and the control group, followed by alcohol dependence syndrome (1.75% > 0.95%; 0.47% > 0.13%).  Figure 2 displays the cumulative incidences of SRDs both in mental disorder and in control groups. The cumulative incidence of SRDs in the mental disorder group increased faster than that in the control group and reached 5 times higher by the end of year 2009 (*p* < 0.05).

Table 3 shows the result of the multivariable Cox proportional hazard regression model, an adjusted HR of 5.09 (95% CI: 4.74-5.48), suggesting that the risk of subsequent onset of SRDs in persons with selected mental disorders is about five times higher than that in those without after controlling for age, sex, and urban and income levels. Across the different diagnoses of selected mental disorders, patients with personality disorders were at the highest risk (HR=25.05, 95% CI=14.37-43.67), followed by schizophrenia (HR=11.17, 95% CI=7.73-16.14), affective psychoses (HR=10.99, 95% CI=9.36-12.90), adjustment reaction disorders (HR=9.67, 95% CI= 6.97-13.51), and neurotic disorders (HR=4.84, 95% CI= 4.48-5.23). Adjusted HRs for age, sex, and urban and income levels appear significant. Compared to the elderly age group (≧70 years), all other age groups had higher risks, particularly the age groups in the 20-49-year range, where hazard ratios (HR) ranged from 5.61 (95% CI= 4.48-5.95) to 7.92 (95% CI=6.89-9.11). People aged 30-39 had an almost 8-fold higher risk for developing SRDs than the elderly. Males had over a 4-fold higher risk (HR= 4.30; 95% CI=4.04-4.57) than females. People living in the lowest urbanization areas (HR= 1.30; 95% CI=1.19-1.43) showed the highest risk for developing SRDs. Compared to persons in the highest income level, those in every lower income level were at significantly higher risks for having SRDs. Individuals in the lowest income level (≦NT$ 18,780) showed the highest HR.

Furthermore, when we analyzed subsamples stratified by age, sex, and urban and income levels by using multivariable Cox proportional hazard models (Table 4), we found the adjusted HRs were all significantly higher than 1.0, with hazard ratios ranging from 2.12 (95% CI=1.72-2.62) to 14.55 (95% CI=7.89-26.83), including age (10 to 70 years or more), gender (males and females), urban level (lowest to highest urbanization), and income level (≦18,780 to ≧45,801). Among the individuals aged 10-19, those with selected mental disorders had a 14.55-fold higher risk for subsequent SRDs than those without and, interestingly, the hazard ratio increased as age decreased. Males with selected mental disorders had around a 9 times (HR= 8.68; 95% CI=7.35-10.26) higher risk for developing SRDs than those without. For people living in the urbanization levels above moderate, those with selected mental disorders would have around a 6 times higher risk for developing SRDs than those without. When stratified by income level, among individuals at the income level of NT$ 28,801-36,300, those with selected mental disorders showed close to a 7 times higher risk (HR= 6.62; 95% CI: 4.54-9.66) for developing SRDs than those without.

Table 5 shows the estimated hazard ratios of subsequent onset of each subtype of substance-related disorders for each selected mental disorder while accounting for age, gender, urban level, and income level. The results show that, for subsequently developing alcohol-induced mental disorders, the hazard ratio for affective psychoses was the highest (HRs=64.75), followed by personality disorders (HR=38.64), schizophrenia (HR=30.62), and adjustment reaction (HR=12.59). For subsequently developing alcohol dependence syndrome, the hazard ratio for personality disorders was the highest (HR=47.3), followed by affective psychoses (HR=22.10), adjustment reaction (HR=21.56), and schizophrenia (HR=17.82). With regard to the risk of subsequently developing drug-related disorders, those with personality disorders had the highest risk to develop drug-induced mental disorders, drug dependence, and nondependent abuse of drugs (HRs=60.77, 82.40, and 22.63, respectively; Ps < 0.0001). Those with the second high risk of developing drug-induced mental disorders, drug dependence, and nondependent abuse of drugs were affective psychoses (HR=26.88), adjustment reaction (HR=68.02), and schizophrenia (HR=9.37), respectively. However, neurotic disorders had the lowest hazard ratio of developing either alcohol-related or drug-related disorders.

## 4. Discussion

In the present study, we found that individuals with affective psychoses, neurotic disorders, schizophrenia, personality disorders, or adjustment reaction had significantly higher risks for subsequently developing SRDs compared to those without, independent of age, sex, and income and urban levels, suggesting that the prior onset of any one of the above-mentioned mental disorders later on does have potential risks for developing a SRD.

Our findings are consistent with existing studies and support the onset of primary mental disorders prior to the development of substance dependence and/or abuse. Previous studies indicated that anxiety disorders, phobic and panic disorders, personality disorders, antisocial personality disorders, conduct disorders, mood disorders, and schizophrenia were associated with later onset of substance use disorders [5, 15–22, 24–26, 28, 36, 51–55]. Our study found that when compared to their counterparts with no specific mental disorders and regardless of the existence of other comorbid mental disorders along with their specific diseases, persons with personality disorders had the highest risk of developing a SRD (HR=25.05), followed by schizophrenia (HR=11.17), affective psychoses (HR=10.99), adjustment reaction (HR=9.67), and neurotic disorders (HR=4.84). And this order was the same as that found for developing nondependent abuse of drugs—the major subtype in the SRD onsets.

In subsample analyses, we found that, among all age groups, the youngest group of 10-19 years is at the highest risk for those with any specific mental disorders to develop a SRD in their later life. Review articles pinpointed that child psychopathology is associated with early onset of substance use and abuse in later adolescence [56, 57]. In addition, US epidemiological studies showed that mental disorders usually occur at an earlier age than do substance use disorders, and the highest risk of secondary substance use disorders was found among people whose mental disorders begin during either childhood or adolescence [58], and the median time interval between first onset of primary mental disorders and first onset of secondary substance disorders is 5 years or more [58]. A study for the National Institute of Mental Health (NIMH) epidemiologic Catchment Area Program reported that the lifetime prevalence of onset sequencing pattern for any mood disorder occurring first is higher than for any substance use disorder, and at least 75% of comorbid subjects had their first onset disorder before age 20 [26]. In addition, one thing that should be noted is that attention-deficit/hyperactivity disorder (ADHD) in childhood and adolescence has been suggested to be related to subsequent SRD in adulthood [59, 60]. The shortage of subjects aged 10-19 years in this study along with the low reported prevalence rates of ADHD in Taiwan [61], however, led us not to include it in our analyses. Nevertheless, the risk of ADHD for developing a SRD is worth examining in a future study.

By controlling for the studied mental disorders and other confounding variables, we found the risks for developing a SRD were over five times higher for people aged 20-49 years and over two times higher for people aged 10-19 or 50-59 years, compared to the risk for people aged 70 years and older. As the mean duration of the onset of SRDs for people with the specific mental disorders was 2.12 years (SD=2.12), which was significantly (*p* < 0.001) less than 3.28 years (SD=2.00) in people without (median duration: 1.54 and 3.22 years, respectively), we suggest that health practitioners should pay more attention to the possible use of substances with their clients who have mental problems and adopt some strategies for early prevention or prompt treatment.

In our study, males were more likely to develop SRDs than females and the risk was about 4 times higher. This gender difference in substance use or disorders has been found across different age groups and nations [16, 18, 19, 62–64]. Nevertheless, there may exist gender differences in the magnitudes of the risks for developing specific substance use disorders according to different mental disorders [65, 66]. Our results also revealed that the risk of developing a SRD for people living in the lowest urbanization area is 30% higher than the risk for people living in the highest urbanization-level area. This finding is consistent with the findings of Simmons and Havens' study (2007) [46]. We also found that people in lower income levels were at higher risks for developing SRDs, and the risk increased as the income level declined.

Our study found that affective psychoses and personality disorders were at greatest risk for developing alcohol-related disorders. The likelihood of developing alcohol dependence syndrome and drug-related disorders (drug-induced mental disorders, drug dependence, and nondependent abuse of drugs) were also particularly high for patients with personality disorders. Moreover, the high hazard ratios of developing drug dependence for patients with adjustment reaction and of developing nondependent abuse of drugs for patients with schizophrenia are notable. However, these findings suggest an important thing; that is, health professionals in clinical settings should take steps to prevent patients with personality disorders from substance use and abuse although the prevalence of the disorders is not as high as those of major mental disorders such as affective psychoses and schizophrenia.

In addition, the variation in risk for each subtype of substance-related disorders across mental disorders shown in our study results suggests a need for future studies to investigate the relative risks across a broader range of mental disorders for each category of SRDs. Such studies would provide information for different etiological pathways and tailored prevention strategies.

Our study has several strengths. First, to our knowledge, of all the studies on the relationship between mental disorders and SRDs, this study is the one with the largest sample size and study subjects covering individuals in late childhood, adolescence, adulthood, and old age. Second, we excluded subjects with SRDs before the index date, on the index date, and one year after the index date. This was done to ascertain that no subjects had any SRDs prior to the onsets of mental disorders in order to examine the concerned temporal link between the mental disorders of interest and SRDs.

There are some limitations in the present study. First, there is a lack of some information on related biomarkers such as genetic data, body mass index, hormones, education, lifestyles (e.g., physical activity, smoking, drinking, and drug use or abuse), sleep quality, and family medical history (e.g., psychiatric disorders, substance use/abuse). Second, it is impossible to clarify the severity of the diseases among the cases from the data since the cases were identified by ICD-9-CM codes. To ensure the accuracy in identifying actual cases, we restricted the diagnosis of disease to three outpatient visits within one year or a one-time hospitalization. Third, we were unable to access the patient's living address from our database due to Personal Data Protection Law and the data actually provided us the local areas in which beneficiaries enrolled in the health insurance program via their employment systems and so these areas may not be their residing but working areas. However, despite the above-mentioned weaknesses in this study, the strengths of the NHIRD with longitudinal data since 1995 provide a good opportunity for us to establish a temporal relationship between the specific mental disorders and SRDs.

In the present study, we provided evidence on the temporal link between the specific mental disorders and substance-related disorders in which a prior onset of any selected mental disorders could increase the risks for developing SRDs. Due to the limitation of the sampling design in NHIRD, we were unable to trace the disease trajectory for each subject for an entire birth cohort. Therefore, we caution against assuming a causal relationship between these two classified disorders.

## 5. Conclusion

In this population-based cohort study, we found that individuals with selected mental disorders, including affective psychoses, neurotic disorders, personality disorders, and adjustment reaction, had a fivefold higher risk for subsequent onset of substance-related disorders as compared to those without selected mental disorders, and among them, those with personality disorders had the highest risk. The duration from observing a mental disorder to having a later onset of substance-related disorder is around two years, suggesting that health professionals in their practices should pay more attention to the individuals with mental problems from substance use and abuse.

## Figures and Tables

**Table 1:** The distribution of sociodemographic characteristics between mental illness and control cohorts and of the mental illness at baseline.

	Mental Illness		Control		*P*-value
	(N=124,423)		(N=124,423)		
	N	(%)	N	(%)	
Disease category^*¥*^					
Neurotic disorders	114,114	(91.71%)			
Affective psychoses	21,754	(17.48%)			
Adjustment reaction	6,133	(4.93%)			
Schizophrenia	3,297	(2.65%)			
Personality disorders	1,710	(1.37%)			
Age (years)	48.84	(17.10)	48.78	(17.11)	0.436^†^
10-19	4964	(3.99%)	5161	(4.15%)	0.548
20-29	14632	(11.76%)	14517	(11.67%)	
30-39	20091	(16.15%)	20106	(16.16%)	
40-49	26809	(21.55%)	26899	(21.62%)	
50-59	23298	(18.72%)	23158	(18.61%)	
60-69	18124	(14.57%)	18135	(14.58%)	
≥70	16505	(13.27%)	16447	(13.22%)	
Gender					0.708
Male	48529	(39.00%)	48438	(38.93%)	
Female	75894	(61.00%)	75985	(61.07%)	
Urban level					1.000
Highest urbanization	28284	(22.73%)	28281	(22.73%)	
High urbanization	34946	(28.09%)	34943	(28.08%)	
Moderate	21999	(17.68%)	22002	(17.68%)	
Low urbanization	21939	(17.63%)	21931	(17.63%)	
Lowest urbanization	17255	(13.87%)	17266	(13.88%)	
Income level (NT$)					1.000
≦18,780	61421	(49.36%)	61418	(49.36%)	
18,780-22,800	33219	(26.70%)	33209	(26.69%)	
22,801-28,800	7347	(5.90%)	7339	(5.90%)	
28,801-36,300	7723	(6.21%)	7719	(6.20%)	
36,301-45,800	7583	(6.09%)	7576	(6.09%)	
≧45,800	7130	(5.73%)	7162	(5.76%)	

^*¥*^: the denominator for each of the selected MI is 124,423; ^†^ denotes the *P* value for testing the mean difference on age variable between two groups.

**Table 2:** The follow-up profiles between the mental illness and the control groups.

	Mental Illness		Control	
	(N=124,423)		(N=124,423)	
The median duration of the onset of SRD (years)	1.54		3.22	
The mean duration of the onset of SRD (mean, SD)^†^	2.12	2.12	3.28	2.00
The subsequent onset of SRD (N, %)^†^	4244	3.41%	861	0.69%
Alcohol-induced mental disorders	656	0.53%	68	0.05%
Drug-induced mental disorders	426	0.34%	33	0.03%
Alcohol dependence syndrome	1185	0.95%	159	0.13%
Drug dependence	554	0.45%	23	0.02%
Nondependent abuse of drugs	2173	1.75%	588	0.47%
The death (N, %)^†^	10374	8.34%	8583	6.90%
The loss of follow-up (N, %)	212	0.17%	197	0.16%
Follow-up to the end year 2009 (N, %)	109593	88.08%	114782	92.25%

^†^ denotes the differences in the distributions for the variables between two groups were significant (*P* < 0.001).

**Table 3:** Estimated hazard ratio of the subsequent onset of substance-related disorders for mental illness compared to those without, adjusted for age, gender, urban level, and income level (N=248,846).

	**Substance-related disorders**		
	HR^*∗*^	(95% CI)	*P*-value
Mental Illness vs. Control	5.09	(4.74,5.48)	<.0001
Neurotic disorders	4.84	(4.48,5.23)	<.0001
Affective Psychoses	10.99	(9.36,12.90)	<.0001
Adjustment reaction	9.67	(6.92,13.51)	<.0001
Schizophrenia	11.17	(7.73,16.14)	<.0001
Personality disorders	25.05	(14.37,43.67)	<.0001
Age (years)			
10-19	2.54	(2.00,2.99)	<.0001
20-29	6.81	(5.90,7.86)	<.0001
30-39	7.92	(6.89,9.11)	<.0001
40-49	5.16	(4.48,5.95)	<.0001
50-59	2.83	(2.44,3.29)	<.0001
60-69	1.82	(1.55,2.14)	<.0001
≥70	Ref.		
Gender			
Male	4.30	(4.04,4.57)	<.0001
Female	Ref.		
Urban level			
Highest urbanization	Ref.		
High urbanization	1.05	(0.97,1.13)	0.2809
Moderate	1.03	(0.94,1.13)	0.5125
Low urbanization	1.07	(0.98,1.17)	0.1345
Lowest urbanization	1.30	(1.19,1.43)	<.0001
Income level (NT$)			
≦18,780	3.89	(3.34,4.52)	<.0001
18,780-22,800	3.03	(2.59,3.54)	<.0001
22,801-28,800	2.21	(1.83,2.67)	<.0001
28,801-36,300	1.73	(1.43,2.10)	<.0001
36,301-45,800	1.72	(1.42,2.08)	<.0001
≧45,801	Ref.		

^*∗*^ calculated by using the Cox proportional hazard model.

**Table 4:** Estimated hazard ratio of the subsequent onset of substance-related disorders (mental illness vs. control) for subsamples.

	**Substance-related disorders**			
	N	HR^*∗*^	(95% CI)	*P*-value
Age (years)				
10-19	10125	14.55	(7.89,26.83)	<.0001
20-29	29149	8.59	(7.04,10.48)	<.0001
30-39	40197	7.17	(6.14,8.38)	<.0001
40-49	53708	4.47	(3.86,5.17)	<.0001
50-59	46456	3.78	(3.12,4.56)	<.0001
60-69	36259	2.12	(1.72,2.62)	<.0001
≥70	32952	2.18	(1.65,2.87)	<.0001
Gender				
Male	96967	8.68	(7.35,10.26)	<.0001
Female	151879	4.24	(3.90,4.60)	<.0001
Urban level				
Highest urbanization	56565	5.69	(4.82,6.73)	<.0001
High urbanization	69889	5.70	(4.92,6.60)	<.0001
Moderate	44001	5.29	(4.44,6.31)	<.0001
Low urbanization	43870	4.93	(4.14,5.87)	<.0001
Lowest urbanization	34521	3.47	(2.95,4.08)	<.0001
Income level (NT$)				
≦18,780	122839	5.65	(5.10,6.26)	<.0001
18,780-22,800	66428	4.25	(3.71,4.86)	<.0001
22,801-28,800	14686	5.01	(3.63,6.93)	<.0001
28,801-36,300	15442	6.62	(4.54,9.66)	<.0001
36,301-45,800	15159	3.77	(2.78,5.12)	<.0001
≧45,801	14292	3.45	(2.45,4.87)	<.0001

^*∗*^: subsamples were stratified by age, gender, urban level, and income level; model analyses were adjusted for other covariates.

**Table 5:** Estimated hazard ratio of the subsequent onset of each subtype of SRDs for those with the selected mental disorders compared to those without, adjusted for age, gender, urban, and income levels.

**Dependent variable**	** HR**	**(95% CI)**	***p*-value**
Independent variable			
**Alcohol-induced mental disorders**			
Neurotic disorders	8.75	(6.78-11.29)	<.0001
Affective psychoses	64.75	(26.76-156.68)	<.0001
Adjustment reaction	12.59	(3.88-40.90)	<.0001
Schizophrenia	30.62	(7.48-125.31)	<.0001
Personality disorders	38.64	(5.29-282.15)	0.0003
**Drug-induced mental disorders**			
Neurotic disorders	11.47	(7.99-14.47)	<.0001
Affective psychoses	26.88	(13.27-54.48)	<.0001
Adjustment reaction	14.39	(4.47-46.40)	<.0001
Schizophrenia	22.51	(7.07-71.65)	<.0001
Personality disorders	60.77	(8.41-439.17)	<.0001
**Alcohol dependence syndrome**			
Neurotic disorders	6.98	(5.89-8.29)	<.0001
Affective psychoses	22.1	(14.91-32.75)	<.0001
Adjustment reaction	21.56	(7.91-58.82)	<.0001
Schizophrenia	17.82	(7.23-43.91)	<.0001
Personality disorders	47.3	(11.64-192.22)	<.0001
**Drug dependence**			
Neurotic disorders	25.32	(16.19-39.60)	<.0001
Affective psychoses	47.25	(21.05-106.08)	<.0001
Adjustment reaction	68.02	(9.44-490.12)	<.0001
Schizophrenia	64.71	(8.97-466.70)	<.0001
Personality disorders	82.4	(11.45-592.81)	<.0001
**Nondependent abuse of drugs**			
Neurotic disorders	3.75	(3.41-4.13)	<.0001
Affective psychoses	7.07	(5.83-8.58)	<.0001
Personality disorders	6.07	(4.12-8.94)	<.0001
Schizophrenia	9.37	(5.83-15.06)	<.0001
Personality disorders	22.63	(10.59-48.35)	<.0001

## Data Availability

The data are not available because of confidentiality and restriction by law from Ministry of Health and Welfare, Taiwan.

## Abbreviations

SRDs: Substance-Related Disorders
SUDs: Substance Use Disorders
SIDs: Substance-Induced Disorders
UNODC: The United Nations Office on Drugs and Crime
NHIRD: The National Health Insurance Research Database
TNHIP: The Taiwan National Health Insurance Program
CCHIA: The Collaboration Center of Health Information Application
NIMH: The National Institute of Mental Health.
